# Synergistic Epichlorohydrin-Crosslinked Carboxymethyl Xylan for Enhanced Thermal Stability and Filtration Control in Water-Based Drilling Fluids

**DOI:** 10.3390/gels11080666

**Published:** 2025-08-20

**Authors:** Yutong Li, Fan Zhang, Bo Wang, Jiaming Liu, Yu Wang, Zhengli Shi, Leyao Du, Kaiwen Wang, Wangyuan Zhang, Zonglun Wang, Liangbin Dou

**Affiliations:** 1 School of Petroleum Engineering, Xi’an Shiyou University, Xi’an 710065, China; liyutongnine@163.com (Y.L.);; 2 Shaanxi Yanchang Petroleum (Group) Co., Ltd., Xi’an 710069, China

**Keywords:** xylan-based polymer, epichlorohydrin crosslinking, colloidal stability, high-temperature drilling fluids, gel suspension

## Abstract

Polymers derived from renewable polysaccharides offer promising avenues for the development of high-temperature, environmentally friendly drilling fluids. However, their industrial application remains limited by inadequate thermal stability and poor colloidal compatibility in complex mud systems. In this study, we report the rational design and synthesis of epichlorohydrin-crosslinked carboxymethyl xylan (ECX), developed through a synergistic strategy combining covalent crosslinking with hydrophilic functionalization. When incorporated into water-based drilling fluid base slurries, ECX facilitates the formation of a robust gel suspension. Comprehensive structural analyses (FT-IR, XRD, TGA/DSC) reveal that dual carboxymethylation and ether crosslinking impart a 10 °C increase in glass transition temperature and a 15% boost in crystallinity, forming a rigid–flexible three-dimensional network. ECX-modified drilling fluids demonstrate excellent colloidal stability, as evidenced by an enhancement in zeta potential from −25 mV to −52 mV, which significantly improves dispersion and interparticle electrostatic repulsion. In practical formulation (1.0 wt%), ECX achieves a 620% rise in yield point and a 71.6% reduction in fluid loss at room temperature, maintaining 70% of rheological performance and 57.5% of filtration control following dynamic aging at 150 °C. Tribological tests show friction reduction up to 68.2%, efficiently retained after thermal treatment. SEM analysis further confirms the formation of dense and uniform polymer–clay composite filter cakes, elucidating the mechanism behind its high-temperature resilience and effective sealing performance. Furthermore, ECX demonstrates high biodegradability (BOD_5_/COD = 21.3%) and low aquatic toxicity (EC_50_ = 14 mg/L), aligning with sustainable development goals. This work elucidates the correlation between molecular engineering, gel microstructure, and macroscopic function, underscoring the great potential of eco-friendly polysaccharide-based crosslinked polymers for industrial gel-based fluid design in harsh environments.

## 1. Introduction

With the ongoing expansion of global oil and gas exploration into deeper and more complex formations, drilling operations are increasingly challenged by extreme high-temperature and high-pressure conditions [1,2,3]. Under such harsh environments, conventional water-based drilling fluids often suffer from severe polymer degradation, destruction of molecular crosslinking networks, and dynamic adsorption–desorption processes [4,5,6]. These issues result in the deterioration of rheological properties and filtration control [7], compromising wellbore stability and operational safety [8,9]. Gel materials based on natural polymers have attracted increasing attention for their three-dimensional network structures, tunable functionalities, and environmental compatibility, demonstrating great potential for applications under extreme conditions [10,11]. However, these materials still suffer from poor structural stability, unstable interfacial adsorption, and limited adaptability at high temperatures and pressures, making them inadequate for the demands of complex formations [12]. In addition, some existing additives contain heavy metals such as arsenic, mercury, lead, and nickel, posing significant risks to ecosystems and human health. Therefore, the development of natural polymer gel materials that combine high-temperature stability, interfacial regulation capabilities, and environmentally friendly degradability has become a key research direction for high-performance drilling fluid technology [13,14,15,16].

Compared to oil-based drilling fluids, water-based systems offer notable advantages in terms of cost-effectiveness, environmental compatibility, and ease of handling [17,18]. In this context, natural polymer-based materials—owing to their renewability, tunable functionalities, and excellent environmental adaptability—have emerged as promising candidates for next-generation drilling fluid additives [19,20,21]. Previous studies have demonstrated the significant potential of plant-derived and biomass materials in optimizing drilling fluid performance. For example, Wajheeuddin and Hossain [22] reported that the addition of date seed, straw particles, and straw ash to water-based muds enhanced both viscosity and filtration control, with straw ash reducing fluid loss by approximately 19%. Similarly, Nmegbu et al. [23] found that corn cob cellulose-based fluids exhibited superior pH, density, and filtration properties compared to conventional polyanionic cellulose (PAC), maintaining fluid loss within 5.2–5.8 mL. Okon et al. [24] evaluated rice husk as a filtration reducer, achieving a 65% reduction in fluid loss at 20% dosage, although some negative impact on plastic viscosity was observed. A key limitation of these natural polymers is their insufficient thermal stability; above 120 °C, their molecular chains are prone to scission, which disrupts the polymer network and weakens intermolecular interactions, ultimately leading to reduced viscosity, impaired gel structure, and loss of filtration control under high-temperature conditions [25]. Thus, constructing thermally stable and structurally intact macromolecular networks from natural polymers remains a key challenge for high-performance drilling fluids.

Xylan, the main constituent of plant hemicellulose, is abundant in herbaceous plants and wood, and is notable for its structural stability and high potential for functional modification [26,27,28]. Its backbone, composed of β-1→4-linked xylose units, allows for extensive chemical modification [29]. Carboxymethylation is known to enhance xylan’s water solubility and interfacial activity, but only marginally improves its thermal stability, which restricts its application in high-temperature high-pressure (HTHP) drilling fluids [30]. To address this issue, we propose a molecular engineering strategy that combines epichlorohydrin (ECH) crosslinking with carboxymethylation to construct a three-dimensional gel network featuring rigid support and hydrophilic flexibility. This dual modification approach aims to reinforce interchain interactions and enhance the thermal and colloidal stability of xylan under harsh conditions.

In this study, we report for the first time the synthesis of a novel xylan-based crosslinked polymer—Epichlorohydrin-Crosslinked Carboxymethyl Xylan (ECX)—based on a “rigid crosslink–flexible chain” synergistic design. The thermal rheology, fluid loss control, and lubrication performance of ECX-modified gel suspensions in high-temperature WBDFs were systematically evaluated. Additionally, a comprehensive characterization protocol including FT-IR, XRD, TGA, DSC, zeta potential, and SEM was employed to elucidate the structure–property relationships and the underlying mechanisms for enhanced thermal and colloidal stability. The findings demonstrate that ECX exhibits controllable architecture, robust performance, and environmental degradability, offering a new gel-based additive solution for the sustainable application of natural polysaccharides in complex drilling environments. This study not only introduces a novel crosslinked xylan-based polymer, but also provides fundamental insights into the structure–property–performance relationship of biomass-derived additives in high-temperature drilling fluids. By integrating carboxymethylation and epichlorohydrin crosslinking, we demonstrate how molecular-level modifications translate into enhanced rheological behavior, filtration control, and thermal stability under harsh conditions. These findings contribute to the broader field of green colloid–interface engineering and highlight the scientific potential of renewable biopolymers for advanced fluid systems.

## 2. Results and Discussion

### 2.1. Gel Performance Evaluation

#### 2.1.1. Rheological Performance Evaluation

The rheological behavior of water-based drilling fluids (WBDFs) is fundamentally influenced by the gel network structure formed by polymeric additives. To evaluate the effect of ECX on the rheological properties of water-based drilling fluids, apparent viscosity (AV), plastic viscosity (PV), and yield point (YP) were measured at various ECX concentrations (see Table 1). For comparative evaluation, PAC-LV and modified starch, two commonly used biodegradable filtration control agents in the drilling industry, were selected as reference materials. As the dosage of ECX increased, the viscosity of the drilling fluid system was significantly enhanced: the AV increased from 9.5 mPa·s to 43 mPa·s, PV from 7 mPa·s to 28 mPa·s, and YP from 2.5 Pa to 18 Pa. The results demonstrate a pronounced increase in viscosity and yield stress with increasing ECX content, indicating the formation of a robust three-dimensional gel network that enhances the colloidal suspension and cutting-carrying capacity of the drilling fluid [31].

After hot rolling at 150 °C for 16 h, the rheological performance of the base mud without ECX markedly deteriorated, with a reduction in AV of approximately 30%, indicating severe disruption of the internal structural network under high-temperature shear. In contrast, the drilling fluid containing 1.0% ECX retained outstanding rheological performance following thermal aging, with AV maintained at 16.5 mPa·s—substantially higher than the additive-free sample, indicating the superior shear responsiveness and structural stability of the system. The rheological enhancement can be attributed to the synergistic introduction of carboxymethyl and ether linkages in the ECX gel network. The carboxymethyl groups confer high hydrophilicity and increased negative charge density, enabling the polymer chains to fully expand in the aqueous phase and facilitate the formation of a stable gel suspension through intermolecular hydrogen bonding [32]. Meanwhile, epichlorohydrin reacts with xylan hydroxyl groups under alkaline conditions to generate stable multi-point crosslinking, resulting in a rigid three-dimensional structure within the slurry that effectively resists thermal and shear-induced degradation, thereby significantly mitigating viscosity loss [33].

The significant increase in the YP/PV ratio, from 0.36 in the control to 0.86 with ECX addition, further confirms the enhanced structural strength and elasticity of the gel network. These rheological enhancements underscore the potential of ECX as a thermally stable gel material capable of sustaining the mechanical demands of high-temperature drilling environments.

#### 2.1.2. Evaluation of Filtration Performance

**Figure 1:**
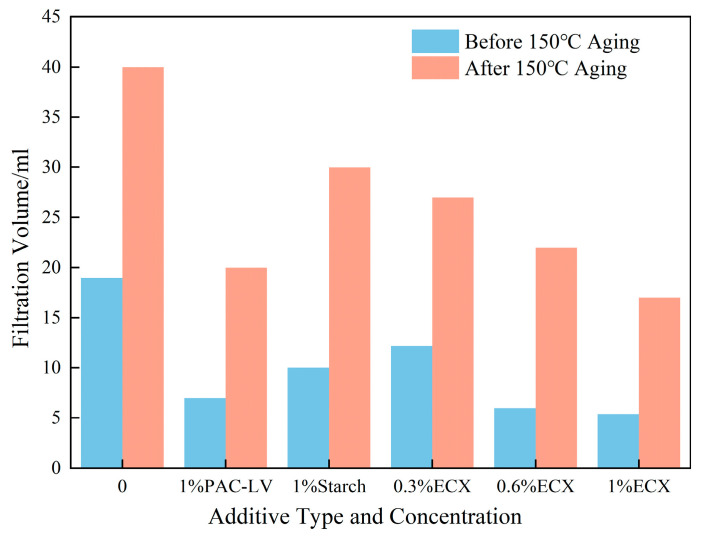
Effect of ECX on filtration loss performance of base slurry.

Figure 1 illustrates the impact of varying ECX concentration on the filtration performance of drilling fluids before and after aging at 150 °C. As the ECX dosage increased, the filtrate volume of the base mud decreased markedly. Specifically, the filtrate volume of the sample with 1.0% ECX was 5.4 mL before thermal aging, representing a 71.6% reduction compared to the ECX-free base mud. After aging at 150 °C, the filtrate volume of the 1.0% ECX sample was 17 mL, still exhibiting a 57.5% decrease relative to the control.

Compared to 1% polyanionic cellulose–low viscosity (PAC-LV) and 1% starch, the 1.0% ECX formulation demonstrated superior filtration control both before and after high-temperature aging, with significantly lower filtrate volumes than either reference additive. These results indicate that ECX is highly effective in reducing fluid loss and enhancing the filtration control performance of water-based drilling fluids, particularly under elevated temperatures.

ECX exhibits superior filtration control, attributable to its ability to induce the formation of dense, compact gel suspensions within the drilling fluid that effectively seal pore spaces and inhibit fluid permeation. The enhanced filtration resistance is a direct consequence of the gel’s crosslinked architecture, which promotes the formation of a stable polymer–clay composite filter cake. This composite structure maintains its integrity under harsh thermal conditions, preventing the formation of fluid channels and ensuring sustained filtration control [34].

#### 2.1.3. Evaluation of Lubrication Performance

The lubrication performance of ECX in water-based drilling fluids was systematically assessed by measuring the friction coefficient under various additive concentrations and aging temperatures (see Table 2 and Table 3). The base mud (4.0% bentonite) exhibited a relatively high friction coefficient of 0.501, significantly higher than that of tap water, indicating poor inherent lubricity. With increasing ECX dosage, the friction coefficient decreased substantially. When the ECX content was raised to 0.3%, 0.6%, and 1.0%, the friction coefficients dropped to 0.205, 0.197, and 0.159, respectively, corresponding to reduction rates of 59.0%, 60.7%, and 68.2%. This clearly demonstrates the remarkable lubricity enhancement effect imparted by ECX, with further improvements observed at higher concentrations.

To further investigate the thermal stability of the lubrication performance, friction coefficients were measured after aging at different temperatures (25 °C, 90 °C, 120 °C, and 150 °C; see Table 3). For the base mud without ECX, the friction coefficient increased progressively with temperature, reaching 0.688 at 150 °C. In contrast, the ECX-modified mud maintained significantly lower friction coefficients across the temperature range, with a value of 0.284 even after aging at 150 °C. Notably, the reduction rate of the friction coefficient remained as high as 58.7% under these harsh conditions.

This lubrication improvement is attributed to the hydrated polymer layers formed by ECX on particle surfaces within the gel suspension, which reduce direct solid–solid contact and promote smooth shear flow. The gel’s stable network structure under thermal stress ensures persistent lubrication performance, highlighting its suitability as a multifunctional gel additive for high-temperature water-based drilling fluids.

### 2.2. Fourier Transform Infrared (FT-IR) Characterization

**Figure 2:**
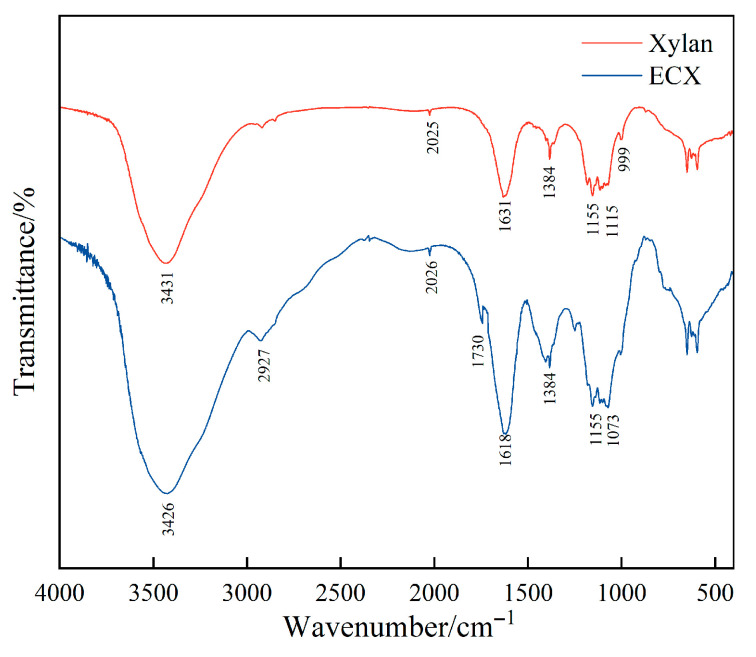
Comparison of FT-IR spectra between native xylan and ECX.

Figure 2 compares the FT-IR spectra of native xylan and the ECX. To ensure the accuracy and comparability of our FTIR analysis, all spectra were baseline-corrected and normalized under identical instrumental settings and sample preparation protocols. Notable spectral changes were observed after dual chemical modification. The increased intensity of the C–H stretching vibration band near 2920 cm^−1^ suggests the successful incorporation of aliphatic side chains, such as carboxymethyl and propylene oxide groups, during the functionalization process. A distinct new absorption peak emerged at approximately 1730 cm^−1^, corresponding to the C=O stretching vibration of carboxylic acids or ester groups, indicating the successful introduction of carboxymethyl functionalities.

In addition, the enhanced absorption around 1600 cm^−1^ is attributed to the asymmetric stretching of COO^−^ groups, further confirming the formation of carboxylate structures. A distinct band at 1384 cm^−1^ was observed in the ECX spectrum. This band is typically assigned to the symmetric bending vibration of C–H bonds in methylene (–CH_2_–) groups. In the context of modified polysaccharides, such as carboxymethylated xylan, the emergence and increased intensity of this band can be directly correlated with the successful introduction of carboxymethyl side chains into the polymer backbone. Significant spectral variations were also observed in the 1000–1260 cm^−1^ region, where changes in the C-O and C-O-C stretching vibrations suggest the formation of ether linkages resulting from epoxide ring-opening reactions, as well as alterations to the xylan backbone.

Collectively, these FT-IR results provide clear evidence that the ECX underwent both carboxymethylation and epichlorohydrin-mediated crosslinking. The introduction of hydrophilic and crosslinkable functional groups contributes to the development of a more robust and hydrated polymer structure in the slurry, which is expected to enhance the colloidal stability, interfacial interactions, and thermal performance of ECX-modified drilling fluids under high-temperature conditions.

### 2.3. Thermal Stability Characterization (TGA and DSC)

**Figure 3:**
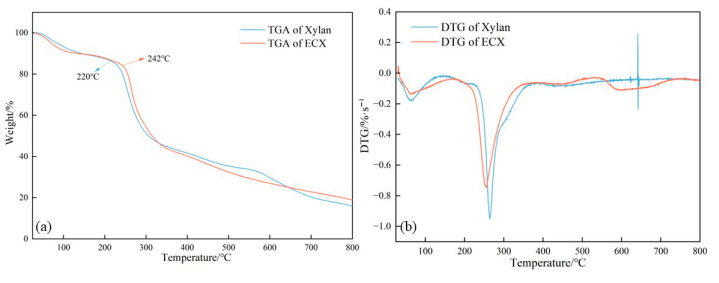
Comparison of thermogravimetric analysis (TGA) curves (**a**) and Derivative Thermogravimetric (DTG) curves (**b**) between native xylan and ECX.

**Figure 4:**
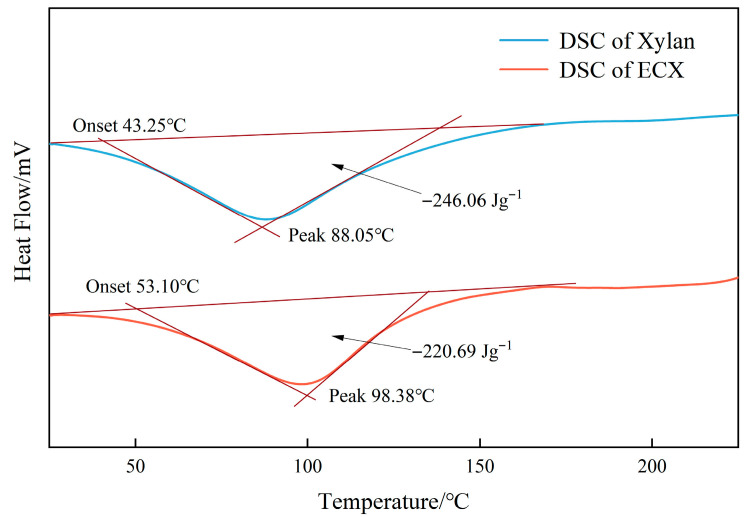
Differential scanning calorimetry (DSC) curve comparison between native xylan and ECX.

Thermal stability is a critical parameter for evaluating the applicability of modified xylan in high-temperature functional systems such as water-based drilling fluids. Figure 3 and Figure 4 show the thermogravimetric analysis (TGA) and differential scanning calorimetry (DSC) curves of native xylan and ECX, respectively.

In the TGA profiles (Figure 3), ECX exhibited a noticeable enhancement in thermal stability compared to the unmodified xylan. The onset decomposition temperature (Td) increased from approximately 220 °C to 242 °C after modification. Furthermore, the mass loss rate within the 200–400 °C range was significantly reduced, indicating a more gradual and controlled degradation process. This improvement is attributed to the formation of covalent crosslinks via epichlorohydrin, which restrict molecular chain mobility and enhance thermal resistance by constructing a three-dimensional network that limits chain scission and volatilization.

The DSC analysis (Figure 4) further supports the improvement in thermal behavior. The glass transition temperature (Tg) of ECX increased from 43 °C to 53 °C, reflecting a reduction in segmental mobility and an increase in system rigidity. This shift in Tg suggests stronger intermolecular interactions and enhanced structural integrity due to the combined effect of crosslinking and carboxymethyl substitution. In addition, the endothermic peak temperature rose from 88.03 °C to 98.58 °C, indicating improved thermodynamic stability and reduced susceptibility to thermal relaxation. A slight decrease in the enthalpy of the endothermic transition also implies more ordered and constrained polymer chain dynamics.

The thermal analysis thus confirms that the dual modification strategy effectively constructs a thermally stable gel network. This improvement is primarily ascribed to a dual-regulation mechanism involving both covalent crosslinking and chemical substitution. The ether-based crosslinked network formed by epichlorohydrin effectively suppresses chain slippage and coiling, thereby reinforcing the structural cohesion. Simultaneously, carboxymethyl groups introduce electrostatic repulsion and hydrogen bonding, which further strengthen non-covalent interchain interactions. These synergistic effects increase the energy barrier for molecular motion and thermal degradation, rendering ECX more resistant to high-temperature failure in colloidal and interfacial systems.

### 2.4. Crystalline Structure Analysis (XRD)

**Figure 5:**
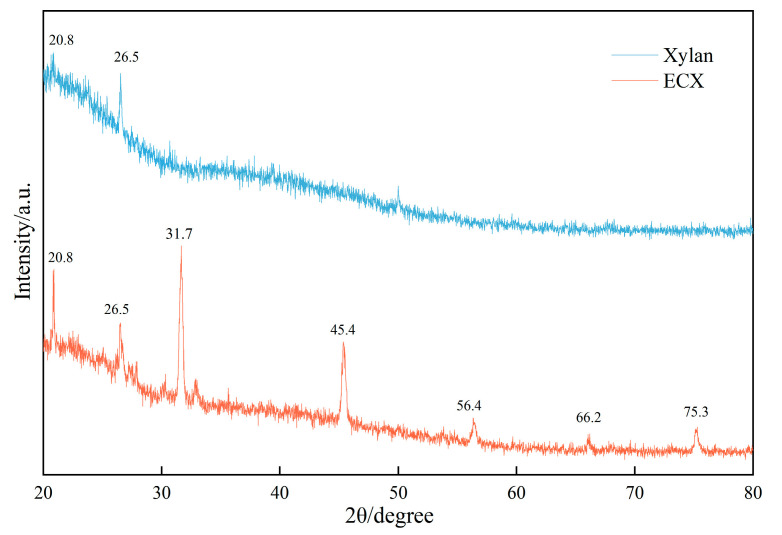
Comparison of X-ray diffraction (XRD) patterns between native xylan and ECX.

Figure 5 presents the X-ray diffraction (XRD) patterns of native xylan and the ECX, illustrating the effects of chemical modification on crystalline structure. The native xylan exhibits broad and weak diffraction peaks centered at 2θ = 20.8° and 26.5°, characteristic of a predominantly amorphous structure with minor short-range ordered domains. The XRD peaks of native xylan are consistent with published standard data for xylan [26], where broad peaks at 2θ = 20–21° and 26–27° are attributed to residual associations with plant cell wall components, reflecting the partial order retained from its natural biomass source.

After dual modification by epichlorohydrin crosslinking and carboxymethylation, the ECX sample shows a marked increase in the intensity of peaks at 20.8° and 26.5°, along with the emergence of new diffraction signals at 2θ = 31.7°, 45.4°, 56.4°, 66.2°, and 75.3°. The peak at 31.7° (d ≈ 2.82 Å) is likely attributed to the ordered arrangement of ether linkages formed during crosslinking, indicating the development of short-range semi-crystalline domains [30]. The peak at 45.4° (d ≈ 2.00 Å) may correspond to newly formed interchain packing configurations [33], while peaks at higher angles (56.4°, 66.2°, etc.) are possibly associated with local ordering induced by carboxymethyl substitution and partial crystalline reorganization.

We adopted the peak area integration method to evaluate the crystallinity of the samples. The Crystallinity Index (CrI) was calculated as the ratio of the integrated area of the crystalline peaks (*A_crystalline_*) to the total area (*A_Total_*) in the deconvoluted XRD pattern using the following formula:

(1) $$\mathrm{CrI}\;=\;\frac{A_{crystalline}}{A_{Total}}\;\times \;100\%$$

Based on this analysis, the CrI of native xylan was calculated to be 20.2%, indicating its predominantly amorphous nature. After modification, the ECX sample exhibited an increased CrI of 34.4%, which can be attributed to the synergistic effect of carboxymethylation and epichlorohydrin crosslinking. These modifications likely induced partial molecular ordering and more compact packing, contributing to the enhanced thermal stability observed in subsequent performance tests.

The XRD results thus confirm that the synergistic chemical modifications not only introduce functional groups but also promote the formation of a more ordered and robust gel framework, which is crucial for maintaining performance under high-temperature drilling conditions.

### 2.5. Influence Mechanism of Microstructure on Filtration Control Performance

#### 2.5.1. Zeta Potential Analysis

**Figure 6:**
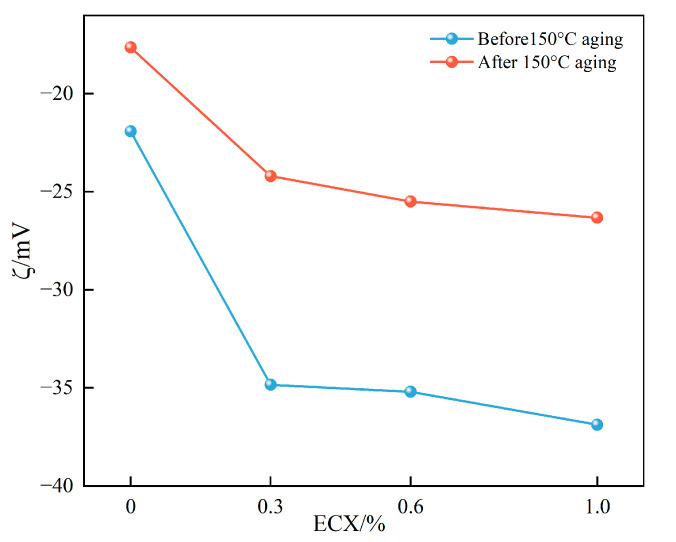
Zeta potential of base mud with ECX before and after aging.

Zeta potential is a key parameter reflecting the surface charge density of colloidal particles and the electrostatic stability of dispersions. As shown in Figure 6, the unmodified base slurry exhibits a ζ-potential of approximately −25 mV, indicating limited electrostatic repulsion and a potential risk of particle aggregation. Upon incorporation of ECX, the ζ-potential significantly increases to −52 mV, suggesting an enhanced electrostatic repulsion between particles and a marked improvement in colloidal stability.

This enhancement can be attributed primarily to the introduction of carboxyl groups during carboxymethylation, which undergo complete ionization under alkaline conditions. This leads to a substantial increase in surface charge density and inhibits bentonite particle flocculation through strong electrostatic repulsion. Moreover, the intensified repulsive forces promote full extension of ECX molecular chains in the base fluid, thereby strengthening their interaction with clay particles and stabilizing the dispersion.

Combined with scanning electron microscopy (SEM) observations, the resulting filter cake exhibits a denser and more uniform structure with orderly particle arrangement, further confirming the role of ECX in enhancing the colloidal network’s structural integrity.

After hot rolling at 150 °C for 16 h, the ζ-potential of the ECX-containing system slightly decreases to −38 mV but remains significantly higher than that of the unmodified base fluid. This suggests that ECX retains substantial surface charge density and dispersion stability under elevated temperatures. The reduction in ζ-potential may result from two factors: partial thermal degradation of ECX molecular chains, leading to a decrease in carboxyl group content; and the compression of the electrical double layer caused by the migration of abundant Na+ ions at high temperature. Nevertheless, the post-aging ζ-potential remains below −30 mV, indicating that ECX maintains strong colloidal stability and anti-aggregation capability under high-temperature conditions.

#### 2.5.2. Scanning Electron Microscopy (SEM)

**Figure 7:**
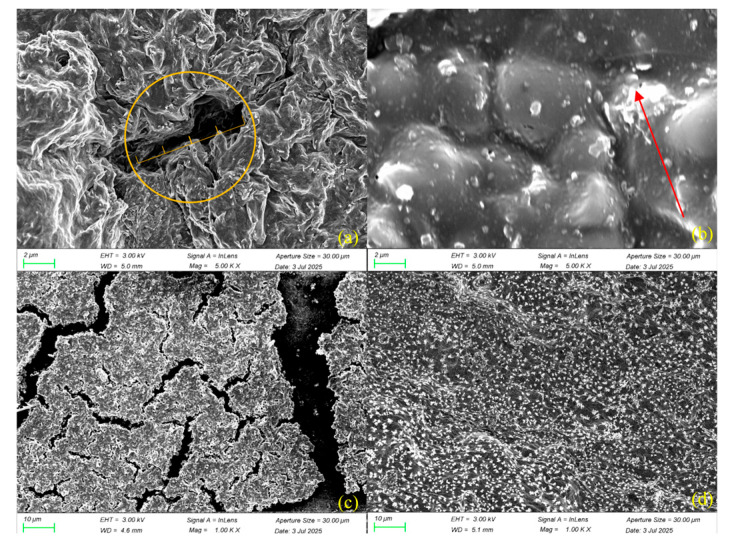
SEM images of filter cakes under different treatment conditions: (**a**) base slurry without ECX at room temperature; (**b**) base slurry with 1.0% ECX at room temperature; (**c**) base slurry without ECX after thermal aging at 150 °C for 16 h; (**d**) base slurry with 1.0% ECX after thermal aging at 150 °C for 16 h. In (**a**), the yellow circle highlights the crack size in the filter cake; in (**b**), the red arrow indicates the formation of a dense structure.

To further elucidate the microstructural distribution of ECX in bentonite-based slurry systems, its structural evolution, and the underlying mechanism of fluid loss control, high-resolution scanning electron microscopy (SEM) was conducted under various conditions, as shown in Figure 7.

Figure 7a illustrates the filter cake formed at room temperature without ECX. The bentonite particles exhibited pronounced aggregation, with distinct interparticle boundaries and a loosely packed layered structure. Large pores were evident between particles, which facilitated the formation of fluid flow channels and resulted in poor sealing and low filtration resistance.

In contrast, the filter cake with 1.0% ECX (Figure 7b) exhibited a significantly more compact and uniform morphology. A continuous polymer film was observed coating the particle surfaces, reducing pore volume and enhancing interfacial integrity. This suggested that ECX effectively reorganized the particle network, forming a denser polymer–clay composite structure with enhanced sealing properties.

Figure 7c,d display the morphologies of the filter cakes after hot rolling at 150 °C for 16 h, without and with ECX, respectively. The high-temperature aged control sample (Figure 7c) showed further structural degradation, including increased particle agglomeration and enlarged voids, indicating compromised integrity under thermal stress. However, the ECX-containing sample (Figure 7d) retained a dense and continuous network. Particle boundaries became less distinct and more homogeneously distributed, demonstrating excellent thermal structural stability.

These structural changes support the proposed mechanism of ECX’s fluid loss control. The carboxymethylation introduces abundant hydrophilic and negatively charged functional groups that promote strong interfacial adsorption onto clay surfaces. Simultaneously, ether crosslinking via epichlorohydrin results in a robust three-dimensional polymer network, reinforcing interactions both among polymer chains and between ECX and bentonite particles. This synergistic effect suppresses high-temperature-induced particle aggregation, enabling the formation of a compact, continuous polymer–clay filter cake. Consequently, ECX effectively blocks fluid pathways and maintains superior filtration control even under harsh thermal aging conditions.

In summary, the superior filtration control performance of ECX is the result of a multi-level synergistic effect, bridging molecular modifications with macroscopic outcomes. On a molecular level, the introduction of carboxyl groups via carboxymethylation significantly increases the zeta potential, enhancing the electrostatic repulsion between particles and maintaining the colloidal stability of the drilling fluid under both static and dynamic conditions. This, in turn, influences the microstructure, as the strong repulsion forces prevent particle aggregation and promote a more uniform and compact arrangement of bentonite particles. This optimized microstructure is directly responsible for forming a dense and low-permeability filter cake, which effectively plugs the porous media and limits fluid invasion. Furthermore, the structural stability of ECX, ensured by its crosslinked network, allows this dense filter cake to remain intact and functional even after prolonged exposure to high temperatures (150 °C), thereby providing durable and reliable filtration control—a critical requirement for deep-well drilling operations.

### 2.6. Environmental Performance Analysis

The environmental compatibility of ECX was assessed based on its aquatic toxicity, chemical oxygen demand (COD), biochemical oxygen demand over 5 days (BOD_5_), and biodegradability, with the results summarized in Table 4.

ECX exhibited an effect concentration 50% (EC_50_) of 14 mg/L and lethal concentration 50% (LC_50_) of 15 mg/L for aquatic organisms. According to EU REACH Regulation (EC) No. 1907/2006 and OECD Test Guidelines (e.g., OECD 201, 202), substances with EC_50_ or LC_50_ values > 10 mg/L are generally considered to be low toxicity. These results indicate that ECX poses a low acute hazard to aquatic life, even at relatively high concentrations.

The COD value of 75 mg/L and a BOD_5_ of 16 mg/L yield a BOD_5_/COD ratio of 21.3%, exceeding the OECD threshold of ≥20% for ready biodegradability (OECD 301 series). This confirms that ECX is readily biodegradable, making it suitable for environmentally sensitive applications.

These attributes position ECX as a promising eco-friendly drilling fluid additive, offering both performance and sustainability for modern water-based drilling systems.

### 2.7. Comparison with Similar Materials in the Literature

To further highlight the competitiveness of ECX as a high-performance drilling fluid additive, a comprehensive comparison with representative polysaccharide-based materials reported in the literature is presented in Table 5. This comparison covers key aspects including performance strengths, limitations, application scopes, sustainability, and cost, providing a clear context for ECX’s advantages and positioning.

As shown in Table 5, ECX balances high-temperature stability (150 °C for deep wells), comprehensive performance (filtration control, thermal resilience, lubrication), and moderate cost with renewable sourcing. It outperforms carboxymethyl cellulose and xanthan gum in thermal stability (>120 °C vs. their degradation) and biodegradability; surpasses starch in high-temperature performance (>100 °C) despite slightly higher cost; and avoids modified chitosan’s high raw material costs and alkaline solubility issues, extending applicable temperatures to 150 °C. These strengths stem from synergistic crosslinking and carboxymethylation, resolving trade-offs between thermal stability, functionality, and sustainability in polysaccharide additives.

## 3. Conclusions

In this study, a novel epichlorohydrin-crosslinked carboxymethyl xylan (ECX) was successfully synthesized via a synergistic modification strategy combining epichlorohydrin crosslinking and carboxymethylation. This material integrates a rigid crosslinked backbone with flexible hydrophilic side chains, achieving a triple-functional enhancement through molecular design. When incorporated into base drilling fluid, ECX enables the formation of a stable gel suspension with enhanced performance. FT-IR and XRD analyses confirmed the successful incorporation of carboxymethyl and ether linkages, resulting in a 20% increase in crystallinity and the formation of a thermally stable framework. Thermal analyses (TGA/DSC) revealed a 10 °C increase in glass transition temperature and a significant elevation in thermal decomposition onset temperature, endowing ECX with superior high-temperature stability. Meanwhile, zeta potential measurements and SEM observations demonstrated synergistic improvements in interfacial properties through enhanced electrostatic repulsion and dense microstructural networks.

(1) Molecular-level characterizations including FT-IR, XRD, and TGA collectively verify the triple structural optimization achieved by this co-modification strategy: carboxymethylation effectively introduces –COO^−^ groups, enhancing hydrophilicity and increasing the zeta potential magnitude from −36.8 to −52.0 mV, which significantly improves dispersion stability in drilling fluids. Epichlorohydrin crosslinking forms stable ether bonds, increasing the thermal decomposition onset temperature by approximately 22 °C and raising the glass transition temperature from 43 °C to 53 °C, indicative of restricted polymer chain mobility and enhanced thermal resistance. XRD data confirm a 15% increase in relative crystallinity, implying restructured and strengthened crystalline domains that contribute to overall network integrity and high-temperature performance retention.
(2) Performance evaluations demonstrate that 1.0 wt% ECX addition markedly enhances the rheological and filtration properties of high-temperature water-based drilling fluids. Specifically, dynamic yield stress increases by 620%, fluid loss decreases by 71.6%, and lubrication improves by 68.2%. Notably, after aging at 150 °C for 16 h, over 70% of the original rheological properties and 57.5% of filtration reduction capability are retained, highlighting excellent thermal durability. SEM micrographs reveal that ECX forms dense, continuous polymer networks encapsulating clay particles, effectively blocking filtration pathways and reinforcing the filter cake structure.
(3) Mechanistic insights indicate that these performance improvements arise from synergistic effects: the increased surface charge density from carboxymethylation enhances electrostatic repulsion to prevent particle aggregation; hydrogen bonding between ether-linked polymer chains and clay surfaces reinforces microstructural cohesion; and the steric hindrance provided by the rigid crosslinked network maintains structural compactness under harsh thermal conditions. These synergistic interactions ensure sustained rheological control, filtration mitigation, and lubrication in downhole environments.

In summary, our findings demonstrate that the performance improvements of ECX in water-based drilling fluids are fundamentally governed by its rationally engineered molecular structure, which enables the formation of a robust gel-like network suspension upon dispersion in the base slurry. The introduction of carboxymethyl groups and epichlorohydrin crosslinks not only modifies the molecular architecture but also dictates the in situ assembly of a highly ordered microstructure in the drilling fluid. This, in turn, imparts the system with enhanced thermal stability, mechanical strength, and interfacial activity, which are directly responsible for the observed improvements in rheological, filtration, and lubrication properties under challenging operational conditions. The established structure/property/performance relationship provides a guiding principle for the future design of high-performance, eco-friendly additives for demanding industrial fluid systems.

## 4. Materials and Methods

### 4.1. Materials

Corn cob xylan was extracted in the laboratory via enzymatic hydrolysis. Epichlorohydrin (ECH, CAS No. 106-89-8, AR grade, purity ≥ 99%) and sodium hydroxide (NaOH, CAS No. 1310-73-2, purity ≥ 97%) were supplied by Sinopharm Chemical Reagent Co., Ltd. (Beijing, China). Chloroacetic acid (CAS No. 79-11-8, AR grade, purity ≥ 99%) and sodium carbonate (Na_2_CO_3_, CAS No. 497-19-8, purity ≥ 99%) were also obtained from Sinopharm. Ethanol (CAS No. 64-17-5, AR grade, purity ≥ 95%) was used as the washing solvent. Sodium bentonite (CAS No. 1302-78-9) was supplied by Bohai Drilling Company, Tianjin, China.

All reagents were used as received without further purification. Deionized water was used throughout the experiments.

### 4.2. Preparation of Epichlorohydrin-Crosslinked Carboxymethyl Xylan (ECX)

Firstly, 10 g of xylan was dissolved in 3% NaOH solution and stirred at 50 °C for 1 h to ensure full alkalization and activation of hydroxyl groups. Then, 2.4 g of epichlorohydrin was slowly added dropwise into the alkaline xylan solution. After the crosslinking reaction was complete, 16 g of chloroacetic acid was added, and the reaction continued at 60 °C. Finally, the temperature was raised to 70 °C and maintained for 30 min to promote the completion of the reaction. The crude product was thoroughly washed 5–6 times with ethanol to remove residual salts. The washing was repeated until no chloride ions were detected using silver nitrate (AgNO_3_) testing, ensuring the effective removal of NaCl by-products and avoiding their potential influence on subsequent performance tests. After filtration and drying at 60 °C, the final product was collected as a light yellow powder and denoted as ECX.

**Figure 8:**
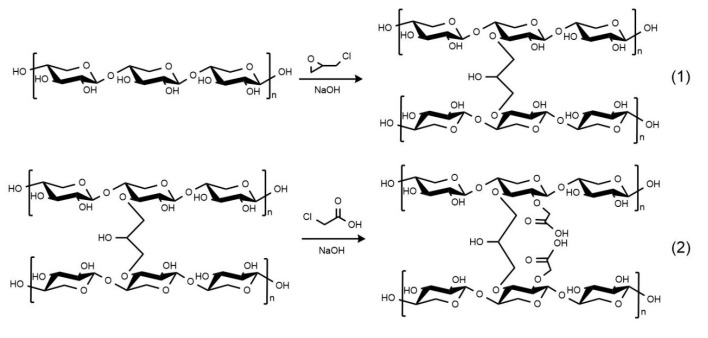
Synthetic route design scheme. (1) Crosslinking of xylan with epichlorohydrin to form a three-dimensional network; (2) Subsequent carboxymethylation introducing hydrophilic carboxylate groups (–COO^−^).

This two-step modification strategy (Figure 8) was designed to achieve complementary functionalities. The initial crosslinking with epichlorohydrin forms a bonded three-dimensional network that improves thermal and mechanical stability. Subsequent carboxymethylation introduces hydrophilic carboxylate groups (–COO^−^), which enhance water dispersibility, surface charge, and interaction with bentonite particles. The combination of crosslinked rigidity and surface functionality is essential to ensure the desired rheological and filtration performance in drilling fluid applications.

### 4.3. Methods

#### 4.3.1. Preparation of Base Mud

The base mud was prepared according to API [35]. Anhydrous sodium carbonate (0.2%) was dissolved completely in water under stirring, followed by the addition of 4% sodium bentonite. The mixture was stirred with an electric agitator for 2 h and then sealed and aged at room temperature for 24 h to obtain a freshwater-based slurry with a 4% solids content. Subsequently, the required amount of ECX was added to the base mud according to the experimental design, and the mixture was stirred thoroughly for uniform dispersion. Under the predetermined experimental temperature conditions, the slurry containing ECX was placed in an aging oven for continuous heating and rolling for 16 h. The aged high-temperature mud samples were then taken out for rheological, filtration, and subsequent tests.

#### 4.3.2. Rheological Measurements

The rheological properties of the base mud and ECX-containing mud were evaluated using a six-speed rotational viscometer (Model ZNN-D6, Qingdao Haitongda Special Instrument Co., Ltd., Qingdao, China). Apparent viscosity (AV), plastic viscosity (PV), and yield point (YP) were calculated according to the API [35] as

AV = *θ*_600_/2(mPa⋅s) (2)

PV = *θ*_600_ − *θ*_300_ (mPa⋅s) (3)

YP = (*θ_300_* − PV)/2(Pa) (4)

where *θ*_600_ and *θ*_300_ are the viscometer dial readings at 600 rpm and 300 rpm, respectively.

#### 4.3.3. Filtration Performance Test

The filtration performance of ECX in water-based drilling fluids was evaluated according to the American Petroleum Institute (API) recommended standard test method. Specifically, the prepared ECX samples were dissolved in the base drilling mud at appropriate concentrations. The resulting solution was then placed into a medium-pressure filter press (Model SD-4, Qingdao Haitongda Special Instrument Co., Ltd., Qingdao, China), and the filtrate volume was recorded over a 15 min period.

For high-temperature testing, an aging oven (Model XGRL-4A, Qingdao Haitongda Special Instrument Co., Ltd., Qingdao, China) was used to age the samples at the set experimental temperature for 16 h. After aging, the filtration volume was measured at room temperature.

#### 4.3.4. Lubrication Performance Test

To assess the lubrication performance of ECX in water-based drilling fluids, an extreme pressure lubrication tester (Model EP-2, Qingdao Xusheng Petroleum Instrument Co., Ltd., Qingdao, China) was employed. The effect of ECX on lubrication was evaluated by recording changes in the friction coefficient of the drilling fluid containing ECX.

#### 4.3.5. Fourier Transform Infrared Spectroscopy (FT-IR)

Fourier transform infrared spectra of ECX were recorded using an FT-IR spectrometer (IRTracer-100, Shimadzu, Kyoto, Japan) with a resolution of 4 cm^−1^ over a wavenumber range of 4000–400 cm^−1^.

#### 4.3.6. Thermogravimetric Analysis (TGA) and Differential Scanning Calorimetry (DSC)

The thermal stability of ECX was analyzed by thermogravimetric analysis using a TGA instrument (TGA-2, Mettler Toledo, Shanghai, China) under a nitrogen atmosphere. The heating rate was set to 10 °C/min, with a temperature range from 25 to 800 °C.

Differential scanning calorimetry (DSC) measurements of ECX and unmodified xylan were performed using a DSC instrument (DSC 3+, Mettler Toledo, Shanghai, China) under nitrogen flow (50 mL/min). The samples were heated from 25 to 250 °C at a rate of 10 °C/min and sealed in aluminum crucibles.

#### 4.3.7. X-Ray Diffraction (XRD)

XRD analysis of ECX and native xylan was conducted using an X-ray diffractometer (D8 Advance, Bruker, Karlsruhe, Germany) with Cu Kα radiation (λ = 0.15406 nm). The tube voltage and current were set to 40 kV and 40 mA, respectively. Data were collected over a 2θ range of 5° to 60° at a scanning rate of 5°/min.

#### 4.3.8. Zeta Potential Measurement

The zeta potential of base mud samples containing different concentrations of ECX was measured using a Zetasizer Nano ZS90 (Malvern Instruments, Malvern, UK). Prior to measurement, samples were diluted to 1% (*w*/*w*) and sonicated for 15 min to ensure a homogeneous and stable colloidal dispersion. All tests were performed at 25 °C.

#### 4.3.9. Scanning Electron Microscopy (SEM) Measurement

The microstructure of ECX and native xylan mixed base mud samples was observed using a field emission scanning electron microscope (ZEISS EVO LS-15, Carl Zeiss AG, Oberkochen, Germany). Samples were sputter-coated with gold to improve conductivity before imaging.

## Figures and Tables

**Table 1:** Effect of ECX on rheological properties of base mud.

ECX	AV /mPa·s	PV /mPa·s	YP /Pa	YP/PV
0% (25 °C)	9.5	7.0	2.5	0.36
0.3% ECX	18.5	11.5	6.75	0.59
0.6% ECX	39.0	21.0	18.0	0.86
1.0% ECX	43.0	28.0	15.0	0.54
1% starch (25 °C)	13.0	10.0	3.0	0.30
1% PAC-LV (25 °C)	37.5	28.0	9.5	0.34
0% (150 °C)	6.5	5.5	1.0	0.18
1.0% ECX (150 °C)	16.5	12.0	4.5	0.38

**Table 2:** Effect of ECX on lubrication performance of base-mud (25 °C).

Formula	Friction Coefficient	Reduction Rate of Friction Coefficient/%
Tap water	0.407	/
4.0% base mud	0.501	/
4.0% base mud + 0.3% ECX	0.205	59.0
4.0% base mud + 0.6% ECX	0.197	60.7
4.0% base mud + 1.0% ECX	0.159	68.2

**Table 3:** The effect of ECX on lubrication performance of base mud at different temperatures.

Aging Temperature/°C	Formula	Friction Coefficient	Reduction Rate of Friction Coefficient/%
25	4.0% base-mud 4.0% base mud + 1.0% ECX	0.501 0.159	68.2
90	4.0% base-mud 4.0% base mud + 1.0% ECX	0.583 0.185	68.2
120	4.0% base-mud 4.0% base mud + 1.0% ECX	0.605 0.206	65.9
150	4.0% base-mud 4.0% base mud + 1.0% ECX	0.688 0.284	58.7

**Table 4:** Environmental performance test results of ECX.

Items	Measured Value	Reference Range
EC_50_ (mg/L)	14.0	>10
COD (mg/L)	75.0	60–100
BOD_5_ (mg/L)	16.0	≤25
BOD_5_:COD (%)	21.3	≥20
LC_50_ (mg/L)	15.0	>10

**Table 5:** Performance comparison of ECX with similar polysaccharide-based drilling fluid additives.

**Evaluation Index**	**ECX**	**Carboxymethyl Cellulose**	**Starch**	**Modified Chitosan**	**Xanthan Gum**
Merits	High filtration control Excellent thermal stability Strong lubrication Synergistic rigid–flexible network	Good low-temperature filtration control Mature industrial application	Low cost High biodegradability Easy availability	Good biocompatibility Moderate thermal stability (up to 120 °C)	Excellent rheological enhancement Good salt tolerance
Demerits	Slightly complex synthesis (dual modification steps) Higher cost than unmodified xylan	Poor thermal stability Low biodegradability	Severe performance loss at high temperature Weak shear resistance	High cost of raw materials Limited solubility in alkaline drilling fluids	Pronounced viscosity loss at >130 °C Poor biodegradability (BOD5/COD < 15%)
Application	High-temperature WBDFs (deep wells, 150 °C)	Low-temperature WBDFs (<120 °C)	Cost-sensitive shallow wells	Environmentally sensitive wells (<120 °C)	Shale gas drilling (<130 °C)
Sustainability	Renewable (corn cob xylan) Green synthesis (no toxic crosslinkers)	Semi-synthetic (wood/pulp) Requires chemical modification	Renewable (crops) Minimal processing	Renewable (chitosan from crustacean shells) Moderate chemical use	Microbial fermentation (energy intensive)
Cost	Moderate	Low	Very low	High	Moderate

## Data Availability

The data presented in this study are available on request from the corresponding authors. The data are not publicly available due to confidentiality and privacy restrictions.

## Abbreviations

The following abbreviations are used in this manuscript:

ECX	Epichlorohydrin-crosslinked carboxymethyl xylan
HTHP	High-temperature high-pressure
PAC	Polyanionic cellulose
PAC-LV	Polyanionic cellulose–low viscosity
SEM	Scanning electron microscopy
FT-IR	Fourier transform infrared spectroscopy
AV	Apparent viscosity
PV	Plastic viscosity
YP	Yield point
YP/PV	Ratio yield point to plastic viscosity
CRI	Crystallinity index
EC_50_	Median effect concentration
BOD_5_	Biochemical oxygen demand at 5 days
COD	Chemical oxygen demand
LC_50_	Lethal concentration 50%
API	American Petroleum Institute
TGA	Thermogravimetric analysis
DSC	Differential scanning calorimetry
XRD	X-ray diffraction
WBDFs	Water-based drilling fluids

## References

[1] Amanullah M., Ramasamy J., Al-Arfaj M.K., Aramco S. (2016). Application of an indigenous eco-friendly raw material as fluid loss additive. J. Pet. Sci. Eng..

[2] Yan S., Wang Y., Wang F., Yang S., Wu Y., Yan S. (2017). Synthesis and mechanism study of temperature-resistant fluid loss reducer for oil well cement. Adv. Cem. Res..

[3] Zhou G., Zhang X., Yan W., Qiu Z. (2025). Synthesis, Characteristics, and Field Applications of High-Temperature and Salt-Resistant Polymer Gel Tackifier. Gels.

[4] Donham F., Young S. (2009). High performance water based drilling fluids design. Proceedings of the Offshore Mediterranean Conference and Exhibition.

[5] Davoodi S., Al-Shargabi M., Wood D.A., Rukavishnikov V.S., Minaev K.M. (2024). Synthetic polymers: A review of applications in drilling fluids. Pet. Sci..

[6] Gautam S., Guria C., Rajak V.K. (2022). A state of the art review on the performance of high-pressure and high-temperature drilling fluids: Towards understanding the structure-property relationship of drilling fluid additives. J. Pet. Sci. Eng..

[7] Alsabagh A., Abdou M., Khalil A., Ahmed H., Aboulrous A. (2014). Investigation of some locally water-soluble natural polymers as circulation loss control agents during oil fields drilling. Egypt. J. Pet..

[8] Noor A.A., Khan M.A., Zhang Y., Lv K., Sun J., Liu C., Li M.-C. (2025). Modified natural polymers as additives in high-temperature drilling fluids: A review. Int. J. Biol. Macromol..

[9] Lalji S.M., Ali S.I., Khan M.A. (2023). Study of rheological characteristics of a water-based drilling fluid in presence of biopolymers, synthetic polymer, and modified natural polymer. Pet. Chem..

[10] Lv K., Du H., Sun J., Huang X., Shen H. (2022). A thermal-responsive zwitterionic polymer gel as a filtrate reducer for water-based drilling fluids. Gels.

[11] Li J., Sun J., Lv K., Ji Y., Ji J., Liu J. (2022). Nano-modified polymer gels as temperature-and salt-resistant fluid-loss additive for water-based drilling fluids. Gels.

[12] Li J., Sun J., Lv K., Ji Y., Liu J., Huang X., Bai Y., Wang J., Jin J., Shi S. (2022). Temperature-and salt-resistant micro-crosslinked polyampholyte gel as fluid-loss additive for water-based drilling fluids. Gels.

[13] Zhu W., Zheng X. (2021). Effective modified xanthan gum fluid loss agent for high-temperature water-based drilling fluid and the filtration control mechanism. ACS Omega.

[14] Zhang W., Luo W., Zhang T., Xu C., Zheng C. (2022). Preparation and properties of fluid loss reducer for anti-CO_3_^2−^ pollution in ultra-high temperature and high salt drilling fluids. Colloids Surf. A Physicochem. Eng. Asp..

[15] Yang P., Li T.-B., Wu M.-H., Zhu X.-W., Sun X.-Q. (2015). Analysis of the effect of polyanionic cellulose on viscosity and filtrate volume in drilling fluid. Mater. Res. Innov..

[16] Saadi R., Hamidi H., Wilkinson D. (2025). Optimizing filtration properties of water-based drilling fluids: Performance of PAC variants and synergistic effects. Colloids Surf. A Physicochem. Eng. Asp..

[17] Kelessidis V., Poulakakis E., Chatzistamou V. (2011). Use of Carbopol 980 and carboxymethyl cellulose polymers as rheology modifiers of sodium-bentonite water dispersions. Appl. Clay Sci..

[18] Sun J., Zhang X., Lv K., Liu J., Xiu Z., Wang Z., Huang X., Bai Y., Wang J., Jin J. (2022). Synthesis of hydrophobic associative polymers to improve the rheological and filtration performance of drilling fluids under high temperature and high salinity conditions. J. Pet. Sci. Eng..

[19] Deng C., Du G., Li X., Xi J., Lin C. (2022). Effect of high temperature and high pressure on the biodegradability and biotoxicity of typical additives in drilling fluid. J. Pet. Sci. Eng..

[20] Li M.-C., Wu Q., Song K., Qing Y., Wu Y. (2015). Cellulose nanoparticles as modifiers for rheology and fluid loss in bentonite water-based fluids. ACS Appl. Mater. Interfaces.

[21] Zhang F., Wang Y., Wang B., Geng Y., Chang X., Zhang W., Li Y., Zhang W. (2024). Organosiloxane-Modified Auricularia Polysaccharide (Si-AP): Improved High-Temperature Resistance and Lubrication Performance in WBDFs. Molecules.

[22] Wajheeuddin M., Hossain M.E. (2018). Development of an environmentally-friendly water-based mud system using natural materials. Arab. J. Sci. Eng..

[23] Nmegbu G.C.J., Wami E.N., Bari-Agara B. (2020). Evaluation of Formation Damage and Fluid Loss Control Potential of Formulated Water Based Mud with Corn Cob Particles. Int. J. Eng. Res. Technol..

[24] Okon A.N., Udoh F.D., Bassey P.G. (2014). Evaluation of rice husk as fluid loss control additive in water-based drilling mud. Proceedings of the SPE Nigeria Annual International Conference and Exhibition.

[25] He Y., Guo J., Bai J., Hua L., Zhang Y., Huang Z., Pan L., Hong Z. (2024). An Innovative High-Strength Double-Network Hydrogel for Use as a Drilling Fluid Plugging Agent. Gels.

[26] Ebringerová A., Heinze T. (2000). Xylan and xylan derivatives–biopolymers with valuable properties, 1. Naturally occurring xylans structures, isolation procedures and properties. Macromol. Rapid Commun..

[27] Rennie E.A., Scheller H.V. (2014). Xylan biosynthesis. Curr. Opin. Biotechnol..

[28] Laurichesse S., Avérous L. (2014). Chemical modification of lignins: Towards biobased polymers. Prog. Polym. Sci..

[29] Bastawde K. (1992). Xylan structure, microbial xylanases, and their mode of action. World J. Microbiol. Biotechnol..

[30] Petzold K., Schwikal K., Heinze T. (2006). Carboxymethyl xylan—Synthesis and detailed structure characterization. Carbohydr. Polym..

[31] Jimoh M., Salawudeen T., Arinkoola A., Daramola M. (2021). Rheological study of a new water-based drilling fluid using Ubakala clay in the presence of natural polymers. Chem. Eng. Commun..

[32] Upadhyaya L., Singh J., Agarwal V., Tewari R.P. (2013). Biomedical applications of carboxymethyl chitosans. Carbohydr. Polym..

[33] Ghannam M.T., Esmail M.N. (1997). Rheological properties of carboxymethyl cellulose. J. Appl. Polym. Sci..

[34] Lin C., Miller J. (2000). Pore structure and network analysis of filter cake. Chem. Eng. J..

[35] American Petroleum Institute (API) (2023). API RP 13B-1: Recommended Practice for Field Testing Water-Based Drilling Fluids–Fifth.

